# Obstetric Antiphospholipid Syndrome From the Perspective of a Rheumatologist

**DOI:** 10.7759/cureus.21090

**Published:** 2022-01-10

**Authors:** Juan Camilo Santacruz, Marta Juliana Mantilla, Igor Rueda, Sandra Pulido, Gustavo Rodríguez, John Londono

**Affiliations:** 1 Spondyloarthropathies Research Group, Universidad de La Sabana, Chía, COL; 2 Rheumatology Department, Universidad Militar Nueva Granada, Bogotá, COL

**Keywords:** hydroxychloroquine, thrombosis, antiphospholipid antibodies, placental insufficiency, obstetric antiphospholipid syndrome

## Abstract

Antiphospholipid syndrome (APS) is an autoimmune disease that can lead to thrombotic or obstetric complications. Recent histopathological studies have shown the absence of placental thrombosis, leading to the consideration of other pathophysiological pathways such as inflammation and complement activation. Due to this, various clinical studies are being carried out with different drug agents in order to avoid their complications. The combination of prophylactic heparin treatment and low doses of aspirin today result in successful pregnancies in most cases. Despite this, a minority of patients require alternative therapies to avoid recurrent miscarriage and decrease obstetric morbidity. Thanks to the better understanding of its pathophysiology, other treatments such as low doses of glucocorticoids, hydroxychloroquine (HCQ), immunoglobulin, pravastatin, and plasmapheresis have been considered in refractory cases, achieving favorable results. Despite the great advances regarding its treatment, unfortunately, there are no treatments with a good level of evidence to reduce late obstetric complications. The evaluation of preconception risk factors, as well as the antiphospholipid antibody profile, is necessary to establish individual risk and thus anticipate possible complications.

## Introduction and background

Antiphospholipid syndrome (APS) is an autoimmune disease associated with the presence of specific autoantibodies, including anticardiolipin (aCL) antibodies, anti-β2-glycoprotein 1 (anti-β2GPI) antibodies, and lupus anticoagulant (aL). These antibodies lead to the generation of thrombotic events and specific obstetric complications [1]. The main target of antiphospholipid antibodies is β2GPI, a plasma protein that avidly binds to phospholipids in cell membranes, even more so when it is dimerized by autoantibody binding, increasing the expression of cell adhesion molecules that ultimately cause a prothrombotic effect. Although APS was originally described as a single entity, a significant difference in treatment for patients with thrombotic or obstetric complications has been established in the last 10 years [2]. This is based on certain observations such as the absence of intravascular or intervillous clots in the placenta of women with antiphospholipid antibodies, IgG fractions with different effects on trophoblast cells in vitro, and a lower risk of recurrent thrombotic events [3-5]. Complications of obstetric APS are divided into those that occur in the first trimester (early complications) and those that occur in the second or third trimester (late complications). These differences are attributed to the change in pathophysiological mechanisms that occur in each gestational period; in the first trimester, the loss of pregnancy is attributed to an inhibitory effect on the proliferation of trophoblastic cells, while in the second and third trimesters, they are a consequence of placental dysfunction [6-8]. In the first trimester, recurrent miscarriage is the most frequent complication of obstetric APS and is observed with a frequency close to 54% [9]. Placental insufficiency can manifest as intrauterine growth restriction, preeclampsia, placental abruption, or a combination of these conditions. Additionally, the antiphospholipid antibody profile appears to have direct implications for gestational morbidity. The aL is the main predictor of adverse outcomes during pregnancy and is the most prevalent serological abnormality, reaching 50.4% of cases [10]. A meta-analysis confirmed that moderate to high aCL antibody titers were associated with the development of preeclampsia and are more prevalent in women with recurrent miscarriages or early pregnancy loss [11]. A recent prospective study showed that anti-β2GPI/human leukocyte antigen-DR complex antibody was frequently associated with recurrent pregnancy loss [12]. Based on all this evidence, aL positivity and high titers of aCL and anti-β2GPI antibodies have major prognostic implications. This review focuses on describing the clinical and pathophysiological characteristics of obstetric APS, as well as the available evidence regarding its treatment to avoid its complications.

## Review

Pathophysiology

**Figure 1:**
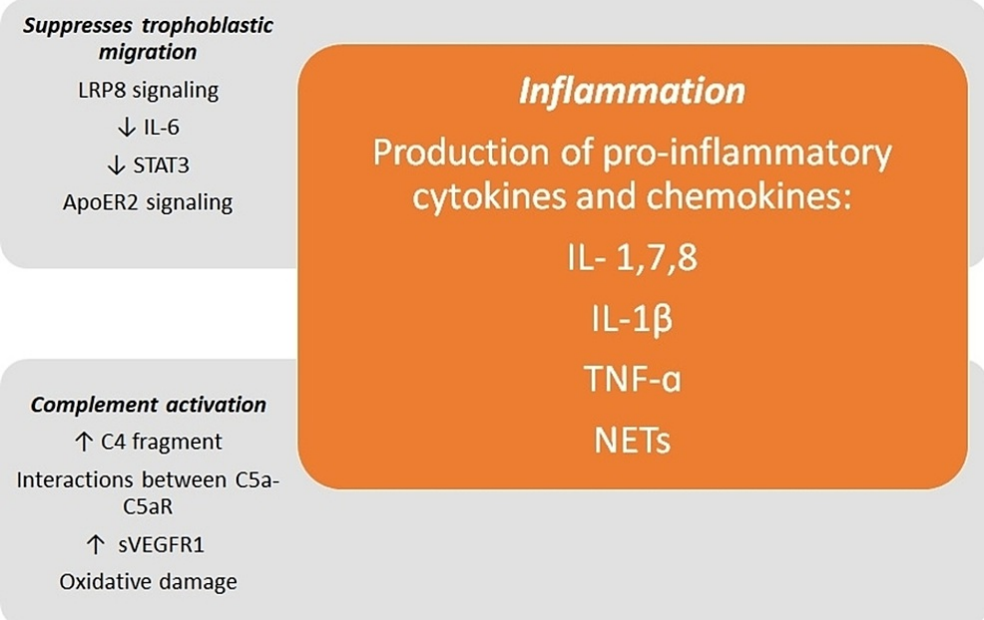
Synopsis of the pathophysiology of obstetric APS ApoER2: apolipoprotein E receptor 2; LRP8: low-density lipoprotein receptor-related protein 8; IL: interleukin; NETs: extracellular neutrophil traps; STAT3: signal transducer and activator of transcription 3; sVEGFR1: soluble vascular endothelial growth factor receptor 1; TNF-ɑ: tumor necrosis factor-alpha References: [20-30]

Antiphospholipid (aPL) antibodies by themselves are considered to cause autoimmune manifestations of obstetric APS and to react against the domain 1 of β2GPI, a ubiquitous glycoprotein involved in the clearance of apoptotic cells and microparticles [13]. In in vitro studies, aPL antibodies inhibit the spontaneous migration of the trophoblast, increase the secretion of soluble antiangiogenic endoglin from the trophoblast, and lead to the loss of trophoblast-endothelium interactions for the conformational change of the spiral artery [14]. These effects are mediated by low-density lipoprotein receptor-related protein 8 (LRP8), which, when activated by the autoantibody cross-linked β2GPI, suppresses trophoblastic migration by reducing IL-6 (promigratory cytokine) levels and STAT3 activity [15]. Recent studies have shown that the limitation of trophoblastic migration by aPL antibodies is mediated by apolipoprotein E receptor 2 (ApoER2), another member of the low-density lipoprotein receptor that interacts with dimerized β2GPI [16]. Decidual invasion by trophoblastic cells depends on the participation of many events, including the response to certain cytokines, the expression of cell adhesion molecules, and the degradation of the basement membrane through protease secretion. It is important to remember that extravillous trophoblasts invade the maternal decidua to anchor the placenta and that the alteration of any of these processes involves the development of placental abruption, fetal loss, preeclampsia, and intrauterine growth restriction [17]. Adequate invasion of the maternal decidua by trophoblasts is very important for the success of pregnancy; in fact, in early pregnancy, if the maternal spiral arteries are not closed properly by endovascular trophoblasts, the strong blood flow of these arteries damages physically or oxidatively the placenta and causes early pregnancy loss [18]. At the end of pregnancy, if the trophoblasts do not transform the maternal spiral arteries into thicker vessels adapted for efficient blood flow, the placenta will not present adequate blood perfusion, leading to an ischemia-reperfusion injury [19]. Additionally, antiphospholipid antibodies localize in the placenta, causing associated inflammatory responses, especially complement activation and neutrophil recruitment, having direct implications in the genesis of placental insufficiency [20]. Furthermore, in vitro studies with human extravillous trophoblasts from the first trimester have shown that anti-β2GPI antibodies trigger the production of pro-inflammatory cytokines and chemokines (particularly IL-1, IL-7, and IL-8) through the Toll-like receptor 4 (TLR4). aPL antibodies induce trophoblasts to secrete IL-1β and IL-8 through the activation of TLR4 and its adapter protein, myeloid differentiation factor 88 (MyD88) [21]. IL-1β is a pleiotropic cytokine that recruits monocytes and neutrophils. Additionally, it activates macrophages, promoting inflammation around trophoblasts and apoptosis [22,23]. Complement activation has also been shown to stimulate the release of tumor necrosis factor-alpha (TNF-ɑ) and soluble vascular endothelial growth factor receptor 1 (sVEGFR1) through leukocyte infiltration, both associated with alterations related to placental implantation and the development of preeclampsia [24,25]. Furthermore, anti-β2GPI antibodies recognize β2GPI bound to the cell surface of neutrophils and stimulate the formation of extracellular neutrophil traps (NETs) through a mechanism dependent on reactive oxygen species and TLR4 [26]. According to in vivo studies carried out in murine models, both complement pathways appear to be involved in fetal damage induced by aPL antibodies [27]. The C4d fragment is a marker of the activation of the classical complement pathway and is present in the placentas of women with systemic lupus erythematosus (SLE) and/or APS with preeclampsia, while it is absent in healthy controls [28,29]. The interactions between C5a and C5aR drive the effector mechanisms of placental injury, including the expression of tissue factors in neutrophils and monocytes, oxidative damage, and the release of sVEGFR1 and TNF [30]. In fact, the presence of high levels of TNF in maternal blood and amniotic fluid of patients with preeclampsia has been confirmed [31]. Figure 1 shows a diagram of the most important aspects of the pathophysiology of obstetric APS.

Diagnosis 

The revised Sapporo criteria incorporating the obstetric clinical manifestations of APS are the most widely used criteria for classifying these patients.

Early obstetric complications

Recurrent Miscarriage

As previously mentioned, recurrent miscarriage is the most common complication of obstetric APS, although it is the least specific. This is the reason why the association should be established when other conditions such as uterine malformations, abnormalities of paternal karyotypes, and maternal endocrinopathies have been ruled out [32]. Additionally, their association will depend on the anti-antibody profile and the titers in which they occur, since they can also tend to be positive in healthy patients or in patients with a history of venous or arterial thrombosis [33].

Late obstetric complications

Derived From Placental Insufficiency and Preeclampsia

Fetal death and preterm delivery as a result of preeclampsia or placental insufficiency are considered to be the most specific clinical features of obstetric APS [34]. A multicenter, case-control study involving cases of fetal death reported positive tests for aPL antibodies in about 10% of cases. After excluding other causes of fetal death, positive results for IgG aCL and IgM aCL antibodies were associated with fivefold and twofold increased odds of fetal death, respectively, whereas IgG anti-β2GPI antibodies were associated with a threefold increased odds of fetal death [35]. Two prospective, observational studies of patients with well-characterized APS found that approximately 10% developed severe preeclampsia despite treatment with heparin and aspirin [36]. Placental insufficiency usually accompanies preeclampsia in APS, although it may occur in its absence [37]. Measurement of reduced blood flow in the uterine arteries by Doppler velocimetry is an indirect indicator of the development of placental insufficiency and/or preeclampsia [38]. Therefore, pregnant women with APS should have an obstetric ultrasound evaluation to assess fetal morphology, growth, and amniotic fluid volume. In the second trimester, Doppler ultrasonography is required to assess end-diastolic blood flow in the umbilical artery. The result of normal end-diastolic flow in the uterine artery at 20-24 weeks of gestation is a strong predictor of good fetal outcomes [39]. Ultrasound evaluation allows establishing signs of placental insufficiency such as fetal growth restriction, oligohydramnios, or abnormal placental vascularization. In fact, a prospective study of 100 pregnancies confirmed that second trimester Doppler ultrasound is the best predictor of late obstetric complications in patients with SLE and/or APS [39].

Risk stratification

A good medical history and a complete antiphospholipid antibody profile should always be taken prior to conception to get an estimate of the potential for both thrombotic and obstetric complications. Risk factors include a high-risk antiphospholipid antibody profile, coexisting autoimmune diseases (particularly SLE), previous thrombotic APS, and complications presenting in previous pregnancies [40]. A meta-analysis demonstrated that triple positivity was associated with a lower probability of having live birth, increased risk of preeclampsia, and having a low birth weight for gestational age. In addition, aL-positive patients were at increased risk of preeclampsia and preterm delivery [41]. The same meta-analysis showed that a history of thrombosis was also associated with a lower likelihood of having live birth, increased neonatal mortality, increased risk of prenatal or postpartum thrombosis, and increased risk of having a newborn with low weight for gestational age [41]. Women negative for aL and with a single positive aCL or anti-β2GPI antibody result have a lower risk of adverse gestational outcomes, as do women with low antibody titers and positive IgM isotype results [42,43].

Treatment

**Figure 2:**
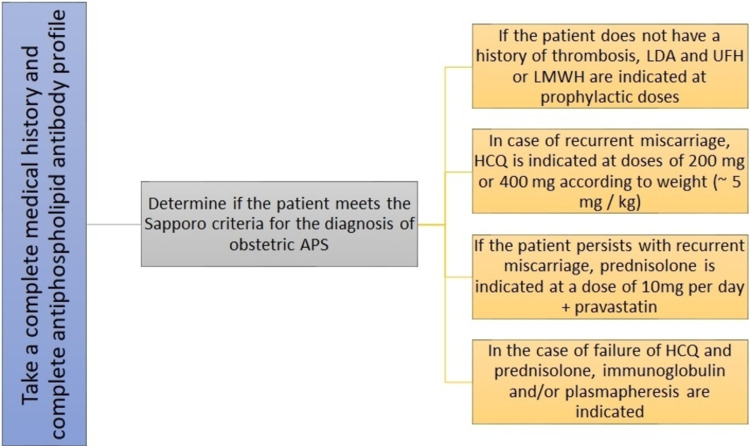
Sequential algorithm for the treatment of obstetric APS and refractory obstetric APS APS: antiphospholipid syndrome; HCQ: hydroxychloroquine; LDA; low-dose aspirin; LMWH: low-molecular-weight heparin; UFH; unfractionated heparin References: [44,48,50,53]

The standard treatment for obstetric APS without a history of thrombosis includes low-dose aspirin (75-100 mg/day) and a prophylactic dose of unfractionated heparin (UFH) or low-molecular-weight heparin (LMWH) [44]. Approximately 70%-80% of pregnant women with APS have had satisfactory results during pregnancy with this therapeutic combination [45]. Some experts prefer to use UFH instead of LMWH based on the results of a 2015 Cochrane review that concluded that their combination can reduce pregnancy loss by 54% [46]. Since the presence of antiphospholipid antibodies increases the risk of pregnancy-induced hypertensive disorders, aspirin is always indicated in the context of their positivity [47]. The treatment options for cases that are refractory (defined as persistence of recurrent miscarriage despite standard treatment) include therapeutic doses of LMWH, low-dose prednisolone (10 mg/day) up to week 14, immunoglobulin, and/or plasmapheresis [48,49]. Immunoglobulin at a dose of 2 g/kg/day monthly and plasmapheresis are high-cost therapies; however, they have been shown to be safe and lead to a high live birth rate in women with little hope of achieving a successful pregnancy. Parenteral treatments should only be considered in the context of standard treatment failure, high-risk profiles, and oral drug failure [50]. Hydroxychloroquine (HCQ) has become the mainstay of treatment for refractory obstetric APS. A retrospective multicenter study of patients with high-risk profile obstetric APS concluded that the addition of HCQ to standard therapy was associated with a higher birth rate [51]. The Hydroxychloroquine to Improve Pregnancy Outcome in Women with Antiphospholipid Antibodies (HYPATIA) study is a multicenter, phase IV clinical trial in which pregnant women with persistently positive aPL antibodies receive HCQ or placebo in addition to standard treatment. The primary endpoint is a combination of aPL-related adverse pregnancy outcomes, including one or more gestational losses and preterm delivery before 34 weeks due to any of the following conditions: preeclampsia, eclampsia, or features arising from placental insufficiency. This study is expected to provide evidence on the effect of HCQ in pregnant women with persistently positive aPL antibodies and thus strengthen its evidence [52]. Due to the similarities in pathophysiology between preeclampsia and atherosclerotic cardiovascular disease, it has been proposed that statins can be used for the treatment or prevention of obstetric complications. In a small study of 11 patients, the benefit of pravastatin at a dose of 20 mg/day with low-dose aspirin and LMWH was evaluated compared with patients treated only with standard therapy. In all patients in the pravastatin group, signs of preeclampsia and placental perfusion remained stable, while in the control group, they progressed [53]. Taking into account the potential role of TNF-ɑ in the development of obstetric complications, a study is being carried out with an anti-TNF (certolizumab) to determine if it is possible to reduce the risk of adverse events in patients with obstetric APS and high-risk antiphospholipid antibody profile (NCT03152058). The evidence is limited on the prevention of recurrent complications related to antiphospholipid antibodies in the second and third trimesters of pregnancy. Additionally, in this context, heparin does not seem to have a great impact on outcomes related to maternal morbidity, although the studies carried out have been insufficiently powered [54,55]. The risk of complications in the carriers of aPL antibodies without obstetric manifestations is not known, although an analysis of a recent European registry of 1,640 cases of women with aPL-related obstetric complications who did not meet the Sydney criteria for obstetric APS had adverse results in pregnancy and benefited from combined therapy with low-dose aspirin and prophylactic LMWH [56]. Since the maternal hypercoagulable state can continue for up to 12 weeks after delivery, patients without a history of thrombosis should receive prophylactic doses of LMWH for 6-12 weeks to reduce the risk of postpartum thrombotic events. Women with thrombotic APS can restart anticoagulation once hemostasis is achieved. Vitamin K antagonists are safe during breastfeeding, but no safety data are available on direct anticoagulants [57]. Table 1 shows the most representative articles for the treatment of refractory obstetric APS, and Figure 2 shows an algorithm of its therapeutic approach.

**Table 1:** Most representative articles for the treatment of refractory obstetric APS aPL: antiphospholipid; HCQ: hydroxychloroquine; HD: hypertensive disorders; IU; international units; LDA; low-dose aspirin; LMWH: low-molecular-weight heparin; PE: preeclampsia

Author and year	Type of study	Drug and dosage	Outcome	Reference
Bramham et al., 2011	Clinical intervention study	Prednisolone at a dose of 10 mg daily	Among 23 pregnancies supplemented with prednisolone, nine women had 14 live births (61%), including eight uncomplicated pregnancies	[48]
Ruffatti et al., 2018	Clinical intervention study	Immunoglobulin at a dose of 2 g/kg/month or plasmapheresis	Parenteral treatments were associated with a significantly higher live birth rate with respect to the oral ones, particularly in high-risk profiles	[50]
Sciascia et al., 2016	Observational, retrospective, single-center cohort study	HCQ at doses of 200 and 400 mg/day	HCQ treatment was associated with a higher rate of live births and a lower prevalence of aPL antibody-related pregnancy morbidity	[51]
Lefkou et al., 2016	Clinical intervention study	Pravastatin at a dose of 20 mg/day	Patients who received both pravastatin and LDA+LMWH exhibited increased placental blood flow and improvements in PE features	[53]
van Hoorn et al., 2016	Clinical intervention study (randomized controlled trial)	Dalteparin 5000 IU by subcutaneous self-administration and daily aspirin 80 mg orally	Combined dalteparin and aspirin treatment started before 12 weeks of gestation in a subsequent pregnancy did not show a reduction of onset of recurrent HD	[54]

## Conclusions

Obstetric APS is a condition that carries great morbidity at any time during pregnancy. However, today, in most cases, full-term pregnancies are achieved with available treatments. Emerging therapeutics also stand out in cases that are refractory given the better understanding of their pathophysiology, focusing on reducing inflammation, aPL antibody levels, and complement activation. All patients require a preconception evaluation, and a risk profile should be established to individualize each treatment. Doppler ultrasound evaluation has become a fundamental tool for both predicting and ruling out late obstetric complications derived from placental insufficiency. Refractory cases that have failed oral medications warrant a multidisciplinary intervention to evaluate parenteral treatments (either monotherapy or in combination), evaluating the cost and available resources. Therapeutic interventions carried out to avoid late obstetric complications are still unknown. The evidence for HCQ in obstetric APS is very promising, pending a full evaluation of the results of ongoing clinical studies. Postpartum, a minimum of six weeks of prophylactic anticoagulation with LMWH is required given the increased risk of thrombosis during this period.
